# Implications of acute temperature and salinity tolerance thresholds for the persistence of intertidal invertebrate populations experiencing climate change

**DOI:** 10.1002/ece3.6498

**Published:** 2020-06-25

**Authors:** Brianna L. Iwabuchi, Louis A. Gosselin

**Affiliations:** ^1^ Department of Biological Sciences Thompson Rivers University Kamloops BC Canada

**Keywords:** acute environmental stress, climate change, emersion temperature, population persistence, sea surface salinity, sea surface temperature

## Abstract

To predict whether populations of marine animals will persist in the face of changing climate conditions, it is informative to understand how past climate conditions have shaped present‐day tolerance thresholds. We examined 4 species of intertidal invertebrates (*Nucella lamellosa, Littorina scutulata, Littorina sitkana,* and *Balanus glandula*) inhabiting the coasts of Vancouver Island, Canada, where the east coast experiences historically warmer sea surface temperature (SST), warmer low tide (i.e., emersion) rock surface temperature (RST), and lower sea surface salinity (SSS) than the west coast. To determine if east coast populations have higher tolerance thresholds to acute stress than west coast populations, animals from 3 sites per coast were exposed to stressful temperatures and salinities in common garden experiments. Emersion temperature tolerance differed between populations only in *N. lamellosa* and *B. glandula*, tolerance thresholds being 1.4–1.5°C higher on the east coast. Water temperature tolerance differed between populations only in *B. glandula* and *L. scutulata* but was highest on the west coast. No differences in salinity tolerance were observed within any species. Thus, there is limited evidence of divergence among east and west coast populations in tolerance of acute stress despite the substantial historical differences in extreme temperature and salinity conditions between coasts. However, based on present‐day summertime SST and RST and known rates of change in these parameters, we predict present‐day tolerance thresholds would be sufficient to allow adults of these populations to tolerate extreme temperatures predicted for the next several hundred years, and that even a slow rate of change in acute tolerance thresholds might suffice to keep up with future temperature extremes.

## Graphical abstract

To determine how past climate conditions have shaped present‐day tolerance thresholds of marine animals, we examined 4 species of intertidal invertebrates inhabiting coasts of Vancouver Island, Canada, which have experienced historically distinct climate conditions. There was limited evidence of divergence among populations in tolerance of acute stress despite the substantial historical differences in extreme temperature and salinity conditions between coasts. However, we predict present‐day tolerance thresholds would be sufficient to allow adults of these populations to tolerate extreme temperatures predicted for the next several hundred years.

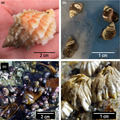

## 1 INTRODUCTION

Recent and predicted future changes in seawater temperature, air temperature, and seawater salinity are significant climate change‐related threats to many marine invertebrates (Byrne, 2012; Wernberg, Smale, & Thomsen, 2012), creating conditions that have the potential to cause alterations in species abundance and distribution (Hawkins et al., 2008). Average global sea surface temperature (SST) has been changing at a rate of 1.1°C per century (IPCC, 2014) and is projected to increase by 0.5–1.8°C by the year 2100 relative to 1986–2005. In turn, sea surface salinity (SSS) trends vary among regions, with certain regions having experienced ocean water freshening by as much as 0.2 PSU from 1950–2008, while other regions became more saline by as much as 0.2 PSU and others yet experienced no significant change (Durack & Wijffels, 2010; IPCC, 2014). It is predicted that SSS will become less saline in high latitude regions that currently have low SSS and more saline in subtropical regions with high SSS (Collins et al., 2013).

Rates of change in global SST and SSS vary by region (IPCC, 2014), such that populations and communities may experience localized trends in climate‐related conditions. One region in which local trends in SST and SSS are well defined is the coast of Vancouver Island, Canada. Yearly SSS minima along the coasts of Vancouver Island have remained unchanged since 1935 (Iwabuchi & Gosselin, 2019). SST along the same coasts, however, is increasing; during summertime, when SST is highest and most stressful for coastal marine animals, SST has been increasing linearly since 1935 at a rate of 0.821–0.967°C per century (Iwabuchi & Gosselin, 2019). Given these changing environmental conditions, for populations to persist in their present‐day range they must either (a) already have broad enough tolerances to function under future environmental conditions or (b) acquire increased tolerance thresholds, through local adaptation and acclimatization, rapidly enough to keep pace with the changing conditions (Clarke, 2003; Sanford & Kelly, 2011). Local adaptation in tolerance thresholds to environmental parameters, such as temperature, is known to occur in coastal marine organisms, even with moderate gene flow among populations (e.g., populations of the marine snail *Chlorostoma funebralis* displaying different thermal tolerances despite evidence of extensive gene flow; Gleason & Burton, 2013) and is hypothesized to be quite common (Sanford & Kelly, 2011), but has not been examined in most marine species. In fact, neither of the above two options are well understood for most coastal marine invertebrates, constraining our ability to predict how populations will respond to future changes in climate conditions. Furthermore, to date studies of local adaptation in tolerance thresholds to environmental parameters by marine species have primarily involved comparisons of populations located across a range of latitudes (Compton, Rijkenberg, Drent, & Piersma, 2007; Fangue, Hofmeister, & Schulte, 2006; Kelly, Sanford, & Grosberg, 2011; Kuo & Sanford, 2009; Leong, Sun, & Edmands, 2018; Morley, Hirse, Portner, & Peck, 2009; Sunday, Bates, & Dulvy, 2010; Sunday et al., 2019; Woolsey, Keith, Byrne, Schmidt‐Roach, & Baird, 2015; Zippay & Hofmann, 2010), an approach that cannot distinguish temperature or salinity effects from latitudinal effects. Comparisons of populations located at a same latitude are thus preferable.

In the context of predicting the effects of future climate change on marine populations, it is informative to understand how past climate‐related conditions have shaped present‐day tolerance thresholds. In particular, the responsiveness of populations to altered climate conditions can be indirectly assessed by examining the extent to which present‐day tolerance thresholds vary among populations of given species relative to existing spatial variation in climate conditions (Bennett, Wernberg, Arackal Joy, de Bettignies, & Campbell, 2015; Sanford & Kelly, 2011; Sorte, Jones, & Miller, 2011). Such intraspecific variation in tolerance thresholds remains poorly understood, likely due to the logistic challenges of such studies. Assessments of the link between interpopulation variation and local environmental conditions are most effective when (a) specimens are collected from two or more populations that are distant enough to have limited gene flow and to experience distinct climates (Bennett et al., 2015), (b) all studied populations are located at a same latitude to avoid confounding latitudinal effects (Bernardo, 1996; Levitan, 2000), and (c) tolerance thresholds of all populations are tested at the same time in a common garden setting using similar methodology (Byrne, 2012).

The southern region of Vancouver Island in British Columbia, Canada, provides an excellent setting to study the relationship between present‐day tolerance thresholds of intertidal invertebrate populations and local environmental conditions. Populations on east and west coasts of the island have experienced persistent (>82 years) regional differences in extreme SST and SSS, with east coast surface waters reaching extreme temperatures that are 5.2°C warmer and extreme salinities that are 8.7 PSU lower during the most stressful months than on the west coast (Iwabuchi & Gosselin, 2019). Correspondingly, the highest daily rock surface temperatures in the intertidal zone during summertime (July–August) low tides are 3.9–4.2°C warmer on the east coast than on the west coast (Iwabuchi & Gosselin, 2019), thus exposing populations of intertidal animals on the two coasts to distinct emersion temperature conditions at low tide. In addition, populations of marine animals on the east and west coasts are separated by dispersal distances >350 km around the south of the island, a substantially greater distance than the average neighborhood size of 10–100 km in marine invertebrates (Palumbi, 2004), suggesting limited gene flow between east and west coast populations. For researchers, however, travel distances by road across the island are only ~150 km, allowing the sampling of intertidal animals from each coast and their return to a common laboratory within a few hours. Furthermore, the coastal waters of the Northeast Pacific are of particular interest due to their high primary productivity, high coastal biomass, and high species diversity (Croom, Wolotira, & Henwood, 1995) which may be threatened by changing climate conditions.

**FIGURE 1:**
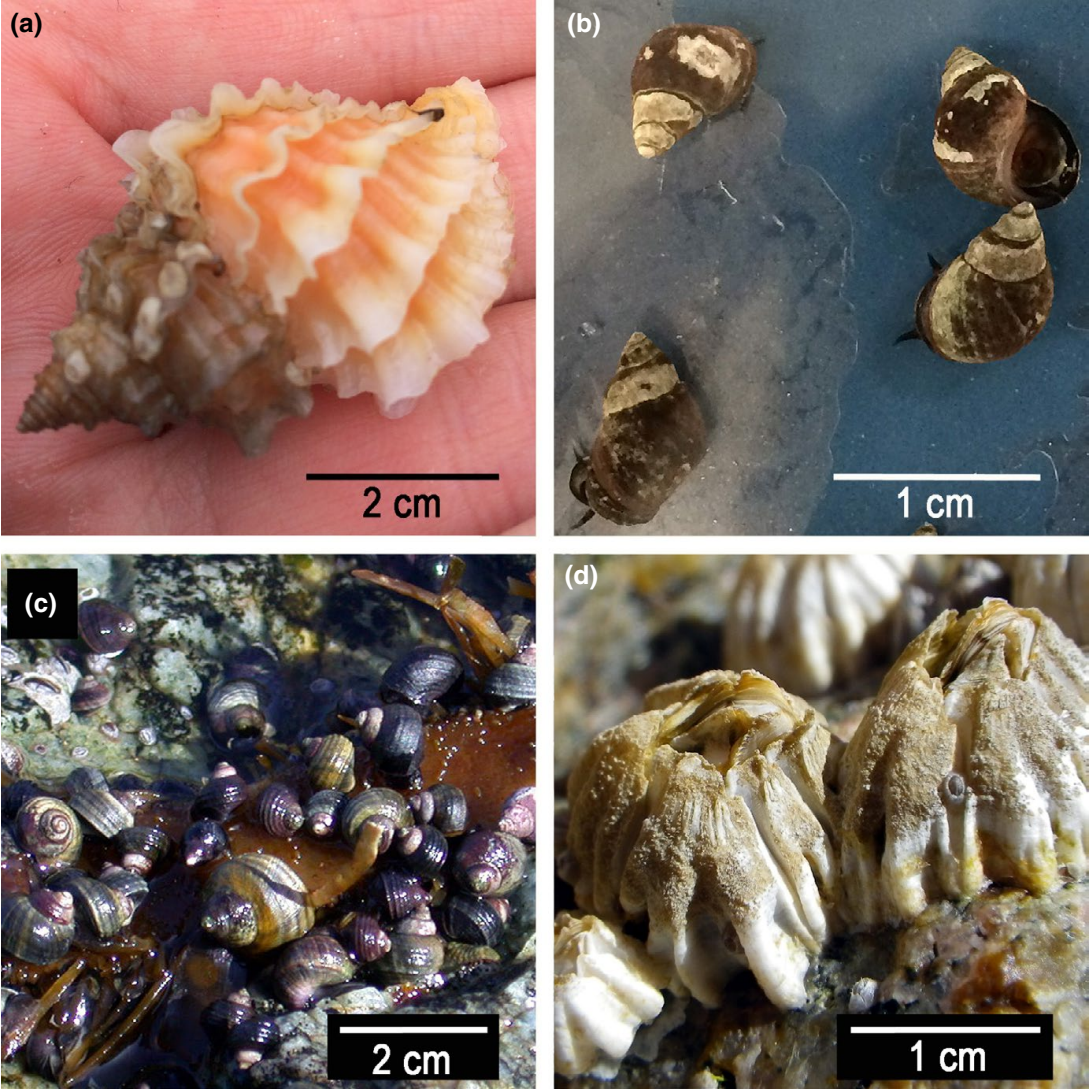
Four species of intertidal benthic invertebrates common to east and west coast Vancouver Island, Canada, including three gastropods: (a) *Nucella lamellosa,* (b) *Littorina scutulata,* and (c) *Littorina sitkana*, and the barnacle (d) *Balanus glandula*

If tolerance thresholds of intertidal species adapt rapidly in response to local SST, SSS, and temperature during low tide emersion, then the persistent and substantial differences in these conditions between the east and west coasts of Vancouver Island, coupled with substantial dispersal distances that restrict genetic mixing of populations, would be expected to have promoted divergence in tolerance thresholds between populations of these two coasts. We therefore hypothesized that east coast populations of marine species should currently exhibit greater tolerance to elevated temperature and to reduced salinity than west coast populations. To test this hypothesis, we examined 4 species of intertidal invertebrates that have substantial populations on both coasts of Vancouver Island: the marine snails *Nucella lamellosa, Littorina scutulata* and *Littorina sitkana,* and the barnacle *Balanus glandula* (Figure 1). As in many benthic invertebrate species, generation time in 3 of these species is relatively short, with individuals starting to reproduce after only 1 year in *L. sitkana* (Reid, 1996), *L. scutulata* (Chow, 1987), and *B. glandula* (Barnes & Barnes, 1956), providing opportunity for rapid evolutionary responses to selective pressures. *N. lamellosa* have a longer generation time, reportedly reaching maturity at 3–4 years of age (Marko, 2004; Spight, 1975). Additionally, these species differ in terms of dispersal abilities, and thus possibly in gene flow: *L. scutulata* and *B. glandula* have dispersing planktonic larvae, whereas *L. sitkana* and *N. lamellosa* have benthic direct‐developing larvae (Strathmann, 1987). Dispersal ability is of particular interest for studies of interpopulation variation, as local adaptation might be more likely in species with limited dispersal capability (Endler, 1977; Foden et al., 2013; Scheltema, 1971). Lastly, these species also differ in terms of habitat preference, with *L. sitkana*, *L. scutulata,* and *B. glandula* all occupying the mid‐intertidal zone to high intertidal zone, while *N. lamellosa* inhabits the low to mid‐intertidal zone. The habitat preference of a species is important to consider when studying tolerance thresholds, as the mid‐intertidal zone to high intertidal zone experiences greater temperature and salinity stress than the low intertidal zone (Somero, 2002; Stephenson, 1942). Thus, it is likely that species inhabiting the high intertidal zone will have more robust tolerance to increased temperature and reduced salinity conditions than low intertidal species.

Determining the tolerance thresholds of local populations that have been exposed to different climate conditions for extended periods of time will help understand how intertidal species may respond to future changes in climate conditions. The present study therefore aimed to determine (a) the extent to which populations of intertidal invertebrates, exposed to different levels of temperature and salinity stress over many generations, have diverged in their acute tolerance thresholds to these stresses, and also (b) if present‐day acute tolerance thresholds of intertidal invertebrates might be sufficient to survive future temperature and salinity extremes that are predicted for these coasts. Using a series of common garden experiments, the study specifically compared the responses of populations inhabiting the east and west coasts of Vancouver Island to elevated temperature during low tide emersion, elevated water temperature, and low salinity. In addition, the inclusion of species with direct development as well as species with planktonic larval development provided insight into the influence of dispersal ability on local tolerance thresholds to temperature and salinity conditions.

## 2 MATERIALS AND METHODS

### 2.1 Study sites and animals

**FIGURE 2:**
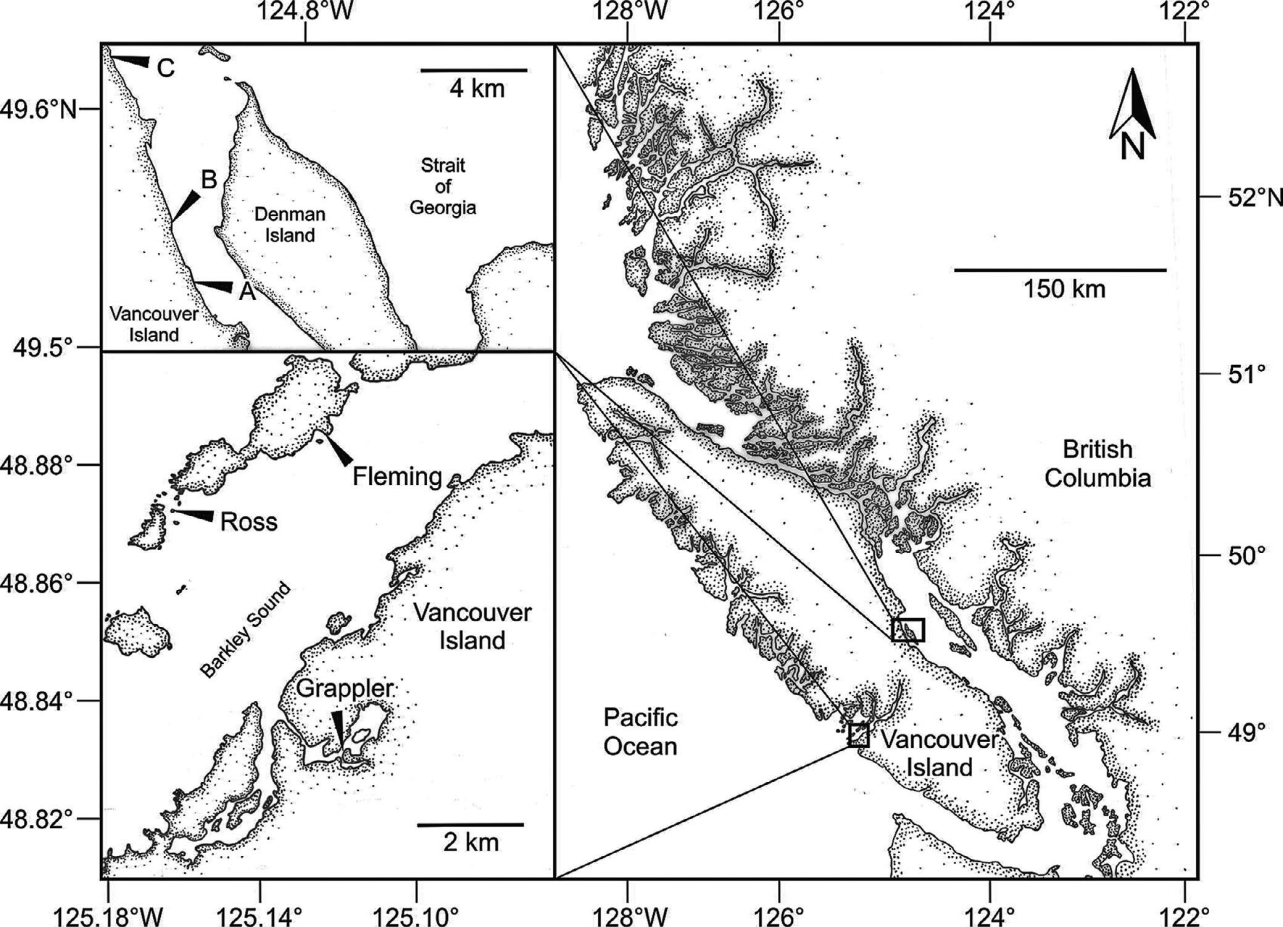
Field sites at which intertidal rock surface temperature was recorded and animal collections were made on the east (top left) and west (bottom left) coasts of Vancouver Island, British Columbia, Canada (right)

For each species of intertidal invertebrate examined in this study, individuals were collected from two populations, one population on the east coast of Vancouver Island (British Columbia, Canada) and the other on the west coast of the island; each population was subsampled from 3 replicate sites per coast, with sites on both coasts being located at similar latitudes. The 3 sites on the west coast of the island were located within Barkley Sound, and the 3 sites on the east coast were located in the Strait of Georgia between Fanny Bay and Royston (Figure 2). Sites within a single coast were 2.5–8.9 km from each other and were selected based on the following criteria: (a) consisting of rocky substrata and (b) experiencing low to moderate wave action. The latter criterion was confirmed by direct observations and by the presence of *Nucella lamellosa*, an intertidal gastropod that does not colonize wave‐exposed habitats (Kitching, 1976). East and west coast sites nevertheless differed somewhat in substratum, being dominated by boulders on the east coast and by bedrock on the west coast, and by tidal amplitude, tides reaching a maximum height of 5.2 m at east coast sites and 3.9 m at west coast sites (Table 1).

**TABLE 1:** Coordinates and characteristics of the intertidal zone at each east and west coast site on Vancouver Island, British Columbia, Canada

Site	Latitude (N)	Longitude (W)	Substrate	Max. tidal height (m)
West coast				
Fleming Island	48°53.07′	125°07.40′	Bedrock & boulders	3.9
Ross Islets	48°52.33′	125°09.72′	Bedrock & boulders	3.9
Grappler Inlet	48°49.91′	125°07.10′	Bedrock & gravel	3.9
East coast				
Site A	49°32.26′	124°51.55′	Boulders & gravel	5.2
Site B	49°33.50′	124°52.30′	Boulders & mud	5.2
Site C	49°36.84′	124°54.15′	Boulders & gravel	5.2

Note

Maximum tidal height refers to the highest high tide recorded in the summer (April–September) of 2015 and 2016 as per chart datum.

Adults of 4 species of rocky intertidal invertebrates were examined: the snails *N. lamellosa*, *L. sitkana*, and *L. scutulata*, and the barnacle *B. glandula* (Figure 1). These species were selected based on the presence of a large number of individuals of the species at all study sites. In addition to experiments determining tolerance thresholds, the upper limit of intertidal distribution of each population was also assessed. At each of the 3 sites per coast, three 5 m wide vertical transects through the intertidal zone were carefully surveyed at low tide; for each species, the highest vertical extent of distribution of live individuals within the transect, relative to chart datum, was measured. Then, for each species, these measurements of upper extent of distribution were averaged among the 3 transects per site, and the averages for the 3 sites per coast were then averaged to obtain a single overall average upper limit of distribution for the population (Appendix S1D).

### 2.2 Field collection of animals

All three common garden experiments in this study were carried out at the Bamfield Marine Sciences Centre (BMSC), on the west coast of Vancouver Island. A separate collection of animals was carried out for each trial of these experiments, with collections at all east and west coast sites being carried out in a 36 hr period. Animals collected on east coast sites were accessed by road and travel time to bring animals from the field to BMSC was 2.0–6.5 hr. West coast sites were accessed by boat, and travel time to BMSC was approximately 2.5 hr. The duration of emersion experienced by all animals was within the timeframe of a low tide emersion period. Heat stress on the trip from east coast sites to BMSC was prevented by placing animals in a cooler containing bags of seawater (11–14°C) as well as ice packs covered by towels; temperature within the cooler always remained below 17°C during transportation, as monitored by Thermochron^®^ iButton temperature loggers (model DS1921G‐F5) placed within the cooler. On the west coast, potential heat stress was minimized by the shorter travel duration and by keeping animals in shaded conditions. Individuals of each snail species (*L. sitkana, L. scutulata* and *N. lamellosa*) and small rocks with at least 10 individual *B. glandula* were collected at each site on days when the daytime low tide dropped below 1.5 m. At the time of collection, adult individuals of each species were collected near the upper limit of the intertidal distribution of the species (Appendix S1D) and were obtained from on (*L. sitkana, L. scutulata* and *B. glandula*) or under (*N. lamellosa*) intertidal rock surfaces that lay outside tide pools.

Upon arrival at BMSC, healthy adults of *N. lamellosa*, *L. sitkana*, and *L. scutulata* were distributed among experimental cages, one species per cage. Experimental cages consisted of plastic containers with screened walls allowing free movement of water through the cage. Ten adult *B. glandula* of a similar size were haphazardly selected on each rock and labeled with a small dot of an oil‐based paint marker on one of their lateral plates. All caged animals were then immediately placed in trays containing aerated seawater filtered to 200 µm, held at 15.0–17.5°C and 30–32 PSU. During all circumstances wherein animals were submerged in seawater (i.e., prior to and during experiments), *N. lamellosa* were held in tanks that were isolated from *L. sitkana*, *L. scutulata,* and *B. glandula*, preventing the exchange of odors and thus stress associated with the proximity of predator and prey.

### 2.3 Tolerance experiments

Three experiments were performed during the summer months (June–August) to compare tolerance thresholds between the east and west coast populations of the 4 study species. These experiments tested population tolerance thresholds to (a) elevated ambient temperature during low tide emersion, (b) elevated water temperature when immersed, and (c) decreased salinity when immersed. Animals were tested as soon as possible after collecting from the field to ensure they were still habituated to the warm summertime conditions experienced in the field; animals were held in the laboratory, unfed, for only 48–72 hr prior to experimentation, to allow them to stabilize after field collection and travel and to ensure they were healthy at the start of the experiments.

#### 2.3.1 Emersion temperature tolerance

To determine tolerance thresholds to emersion temperature, individuals were subjected to a series of species‐specific temperature treatments (Table 2). *L. sitkana* and *L. scutulata* were collected from the 6 study sites on 28 and 29 July 2015; *N. lamellosa* and *B. glandula* were collected on 13 and 14 August 2015.

**TABLE 2:** Summary of emersion temperature, water temperature, and salinity tolerance experimental designs for each of the four species

Species	Emersion temperature tolerance experiment				Water temperature tolerance experiment				Salinity tolerance experiment			
	Replicate cages per site	# indiv. per cage	Total # indiv. used in experiment	Temp treatments (°C)	Replicate cages per site	# indiv. per cage	Total # indiv. used in experiment	Temp treatments (°C)	Replicate cages per site	# indiv. per cage	Total # indiv. used in experiment	Salinity treatments (PSU)
*Nucella lamellosa*	3	8	576	25, 28, 30, 32	4	7	168	25, 28, 29	4	5	120	25, 20, 18, 16, 14, 12, 10, 8, 6, 4, 2
*Littorina scutulata*	5	10	1,500	36, 38, 40, 42, 45	4	10	240	24, 28, 31, 34	5	10	300	25, 20, 18, 16, 14, 12, 10, 8, 6, 4, 2, 0
*Littorina sitkana*	5	10	1,500	36, 38, 40, 42, 45	4	10	240	24, 28, 31, 34	5	10	300	25, 20, 18, 16, 14, 12, 10, 8, 6, 4, 2, 0
*Balanus glandula*	5	10	900	37, 42, 45	4	10	240	24, 28, 31, 34	5	10	300	25, 20, 18, 16, 14, 12, 10, 8, 6, 4, 2

Note

In the case of emersion tolerance experiments, separate groups of animals were placed in each of the temperature treatments, and temperature treatments for a given species were carried out simultaneously. In both the water temperature and salinity experiments, all individuals of a given species experienced all water temperature treatments or salinity treatments expressed in the table (except for those dying before reaching the final experimental treatment). Furthermore, water temperature and salinity treatments were carried out sequentially, starting with the lowest temperature in the case of water temperature experiments, and the highest salinity, in the case of salinity experiments.

Immediately before starting each trial of the emersion temperature experiment, cages (or rocks, in the case of *B. glandula*) were removed from the holding tanks, and residual water was blot‐dried from the animals and cages. Next, the replicate cages (*N. lamellosa*, *L. sitkana*, *L. scutulata*) or rocks (*B. glandula*) were placed in either air‐tight plastic bags or containers that retained high levels of relative humidity and thus minimized desiccation stress throughout the experiment. To ensure high relative humidity, three 4 × 4 cm paper towels saturated with seawater were also added to each bag or container. At the time of sealing the containers, just before moving these to the temperature treatment incubators, sensors (iButton^®^ model DS1923 humidity loggers) inside the containers indicated relative humidity was at least 80%; relative humidity then increased over the following 1–2 hr in all containers, stabilizing at levels of 90%–98%. The bags and containers were transferred into temperature‐controlled incubators, preset to the desired temperature treatment, for a 12 hr duration. The 12 hr duration of these emersion temperature treatments was chosen so as to slightly exceed the maximum duration of emersion conditions during a low tide in the field; the longer duration of treatments in the water temperature and the salinity tolerance experiments, as described below, is consistent with the longer‐term exposure of intertidal animals to seawater in the field.

After the 12 hr treatment, cages or rocks were submerged in aerated seawater at 17°C for a 12 hr recovery period. Animals were then checked for mortality using species‐specific procedures involving the inspection of inactive organisms for movement responses via gentle probing or timed seawater immersion; details of the procedure are listed in Appendix S1A. The temperature treatments used in this experiment where chosen, based on preliminary trials (Appendix S1B) with each species, to ensure mortality outcomes ranging from 0% to 100%. The binomial mortality data (i.e., alive or dead) were used to calculate the temperature lethal to 50% of individuals (LT_50_) for each site and species using general linear model (GLM) analysis in R statistical software (R Core Team, 2015); the LT_50_ values calculated for each of the 3 sites of a same coast were then averaged to represent the population average thermal tolerance.

#### 2.3.2 Water temperature tolerance

Animals used in water temperature tolerance experiments were collected from east and west coast sites on 3 and 4 August 2016, respectively. Cages were distributed among aerated experimental tanks containing 30–32 PSU, 200 µm filtered seawater preheated to a desired temperature treatment. Preliminary water temperature tolerance experiments revealed that all species survived temperatures up to 24°C. To gradually warm the animals up to this temperature, they were exposed to a 1°C increase in seawater temperature per day until 25°C was reached; details of the procedure are listed in Appendix S1C. Then, to determine water temperature tolerances of east and west coast populations, animals from each site were exposed to progressively warmer temperatures, starting at 25°C and increasing at 3°C intervals (Table 2), with one exception: In the last *N. lamellosa* temperature treatment, there was a malfunction of the heater units resulting in a temperature increase of only 1°C from 28°C to 29°C (Table 2). Animals were exposed to a given temperature treatment for 36 hr, followed by an 8 hr recovery period at 17°C, and then a 4 hr mortality check (Appendix S1A) at room temperature (~20°C). Surviving animals were then placed in the next warmer temperature treatment for 36 hr (Table 2). Water temperature treatments for each species ceased when all animals had died. The temperature at death (TAD) of each animal in the experiment was then used to calculate the average TAD for each species, for each site.

#### 2.3.3 Salinity tolerance

East and west coast animals were collected on 22 and 23 June 2016. All seawater used in the experiment, including salinity treatments and recovery periods, was filtered to 200 µm and held at 17–19°C. Reduced salinities in this experiment were obtained by mixing filtered seawater with deionized water. To determine salinity tolerances of east and west coast populations, animals were exposed to 12 progressively decreasing salinity treatments, starting at 25 PSU, then decreasing to 20 PSU, and from then on decreasing at 2 PSU intervals (Table 2). Within each treatment, animals were exposed to a given salinity for 33 hr, followed by a 12 hr recovery period at 30 PSU, and then monitored for mortality over a 3 hr period at room temperature (~20°C). Surviving animals were then placed in the next reduced salinity treatment. The salinity at death (SAD) of each individual animal in the experiment was then used to calculate the average SAD for each species, for each site.

### 2.4 Present‐day tolerance thresholds relative to predicted future conditions

The second goal of the study, to determine if present‐day tolerance thresholds of these intertidal populations might be overwhelmed in the near future by extreme levels of acute temperature and salinity stress, was addressed in two steps. In the first step, present‐day tolerance thresholds to temperature and salinity were compared to the most extreme SST, SSS and emersion temperature conditions recorded on each coast. All data on extreme temperature and salinity conditions for the two coasts were obtained from Iwabuchi and Gosselin (2019). The most extreme (warmest) SST conditions on each coast were identified by seeking the highest summertime (July and August) SST reported for 2006–2016 for each coast. Extreme SSS conditions were defined by the lowest SSS reported from 2006–2016, which on the east coast occurs in June and July, and on the west coast occurs in January and February (Iwabuchi & Gosselin, 2019). Extreme emersion temperatures were defined by the highest intertidal rock surface temperatures recorded at 1.5 m and 2.25 m tidal heights during daytime low tides in the summers (July and August) of 2015–2016 (Iwabuchi & Gosselin, 2019). The next step, assessing whether acute conditions might jeopardize the persistence of these populations in the near future, consisted of estimating how long it would take for extreme conditions of two parameters that are becoming more stressful over time, SST and emersion temperature, to reach levels matching the present‐day tolerance thresholds of each population (i.e., assuming no evolution of tolerance thresholds). Although it is possible that future rates of change in these parameters might differ from present‐day rates, SST has been increasing linearly since monitoring started in 1935 (Iwabuchi & Gosselin, 2019), and thus our calculations of future SST and of emersion temperatures were based on the assumption of continued linear rates of change. This was accomplished by extrapolating forward based on present‐day extreme levels and known rates of change for each parameter, to determine the year when extremes of each parameter would reach the present‐day tolerance threshold of each population. For SST, the rate of change for each coast was based on long‐term (1935–2016) datasets from coastal lighthouse monitoring stations. In contrast, no long‐term dataset exists for intertidal rock surface temperature in this region, so there was no direct way of quantifying rates of change to predict future levels of intertidal substratum temperature. Predictions of future trends in air temperature on these same coasts, however, have been reported (White et. al.,  2016); these trends in air temperature were used to predict future substratum temperature. While air temperature and intertidal substratum temperature are often quite different at any given moment (Judge, Choi, & Helmuth, 2018), the prediction of long‐term rate of change in air temperature was nevertheless used here as a rough estimator of long‐term rate of change in low tide substratum temperature.

### 2.5 Statistical analysis

In the immersion temperature and salinity experiments, each animal was exposed to a series of increasingly stressful conditions and was monitored over time for survival. This allowed us to determine the temperature or salinity at which death occurred for each individual. For those two parameters, tolerance thresholds were quantified using average temperature at death (TAD) and average salinity at death (SAD), with each individual animal being a replicate measure. That approach, however, could not be used in the emersion temperature experiment because the treatment consisted of one emersion period, simulating an extended low tide, and each individual had to be exposed to one treatment condition for the full duration (12 hr); as a result, different individuals were placed in different temperature conditions, and each individual experienced only one treatment. Thus, emersion temperature tolerance thresholds were determined by calculating LT_50_ values. All statistical analyses of data from emersion temperature, water temperature, and salinity experiments, as well as upper limits of intertidal distribution, were completed using R Statistical software (R Core Team, 2015). In each case, data were tested for normality using the Shapiro–Wilk test and for homogeneity of variance using the Fligner–Killeen test. The tolerance thresholds of east and west coast populations to elevated emersion temperature were compared using a general linear mixed model (GLMM) with a binomial distribution (i.e., alive or dead) for each species. In this model, both temperature and coast were designated fixed effects, while site was random. To determine if there were differences in tolerance thresholds to elevated water temperature or reduced salinity between east and west coast populations of a species, TAD and SAD were compared between populations using mixed model nested analysis of variance (ANOVA). In both analyses, coast was treated as a fixed effect whereas site was classified as a random effect and was nested within coast. Finally, to determine the interspecific relationships between upper limit of intertidal distribution and tolerance thresholds (i.e., emersion LT_50_, TAD, SAD), Pearson correlation analyses were performed for each species using the Hmisc package in R.

## 3 RESULTS

### 3.1 Tolerance experiments

#### 3.1.1 Emersion temperature tolerance

**FIGURE 3:**
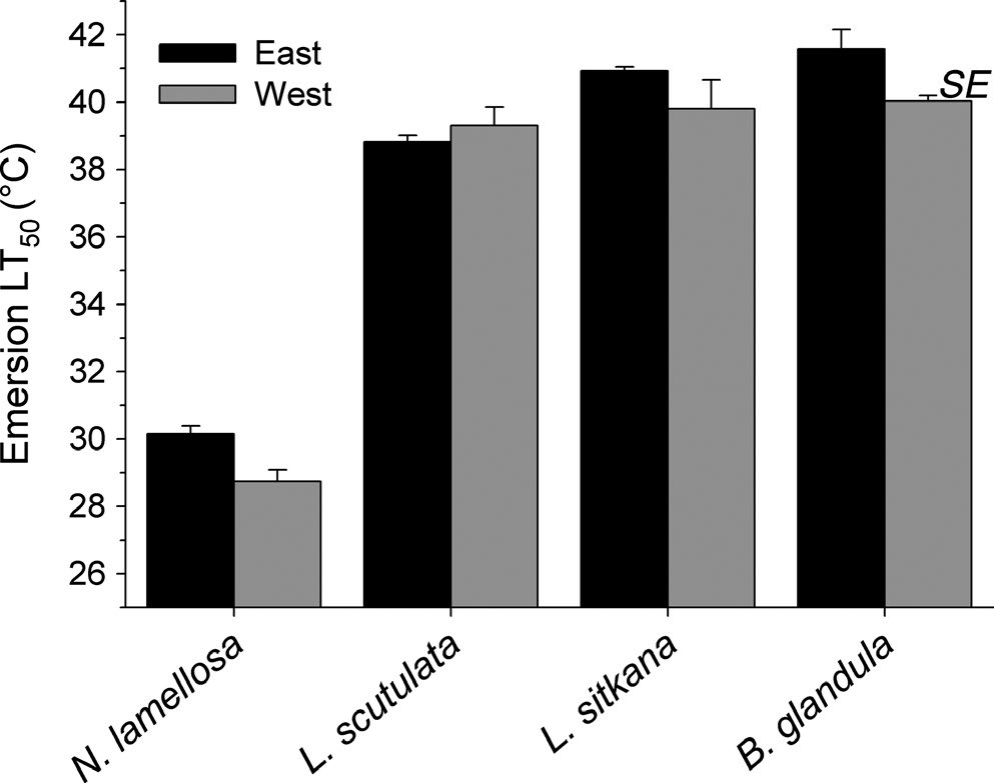
Emersion temperature causing 50% mortality (LT_50_) for the east and west coast populations of four intertidal species

Intraspecific variation in emersion temperature tolerance was detected in 2 of the 4 species; emersion temperature tolerance thresholds differed significantly between east and west coast populations of *N. lamellosa* and *B. glandula,* but not in *L. sitkana* and *L. scutulata* (Table 3). In the 2 species with significant intraspecific variation, east coast populations were more tolerant of elevated emersion temperature than west coast populations; this same trend was also apparent in *L. sitkana* but was not significant (Table 3). For *N. lamellosa*, the LT_50_ of the east coast population was 1.4°C higher than that of the west coast population; in *B. glandula,* the LT_50_ of the east coast population was 1.5°C higher than that of the west coast population (Figure 3).

**TABLE 3:** Results of general linear mixed model (GLMM) with binomial distribution analyzing the effect of location (i.e., east or west coast) on mortality of invertebrate populations in response to emersion temperature treatments

	Estimate	*SE*	Pr(>|z|)
*Nucella lamellosa* ^a^			
Intercept	57.8012	5.5259	<0.001
Temperature	−1.9187	0.1836	<0.001
West coast	−2.2929	0.4291	**<0.001**
*Littorina scutulata* ^b^			
Intercept	51.9691	2.8634	<0.001
Temperature	−1.3349	0.0724	<0.001
West coast	0.6323	0.7117	0.374
*Littorina sitkana* ^c^			
Intercept	56.9818	3.29223	<0.001
Temperature	−1.3889	0.07901	<0.001
West coast	−1.3231	0.87964	0.133
*Balanus glandula* ^d^			
Intercept	47.9282	3.745	<0.001
Temperature	−1.1347	0.08824	<0.001
West coast	−1.9533	0.32441	**<0.001**

Note

Shown are the estimated coefficients, standard errors (*SE*), and statistical significance for the explanatory variables. Each row labeled as West coast in this table represents a comparison with the East coast population.

^a^ 8 animals × 3 replicates × 4 treatments.

^b^ 10 animals × 5 replicates × 5 treatments.

^c^ 10 animals × 5 replicates × 5 treatments.

^d^ 10 animals × 5 replicates × 3 treatments.

Interspecific variation in emersion temperature tolerance was significantly related to the upper limit of intertidal distribution of these species (Table 4). This was primarily due to the low intertidal species (*N. lamellosa)* displaying a considerably lower tolerance (8.7–11.3°C) to emersion temperature than the upper intertidal species (*L. sitkana, L. scutulata, B. glandula)*. No species survived emersion temperatures greater than 42°C (Figure 3).

**TABLE 4:** Pearson correlation analyses of the relationship between upper limit of intertidal distribution of east and west coast populations and tolerance thresholds to elevated emersion and sea surface temperatures and to reduced salinity (*n* = 4)

Parameter	East		West	
	*r*	*p*	*r*	*p*
Emersion LT_50_	.9546	.0454	.9871	.0130
Water temperature at death	.9953	.0045	.9915	.0085
Salinity at death	−.8315	.1685	−.9327	.0673

#### 3.1.2 Water temperature tolerance

**FIGURE 4:**
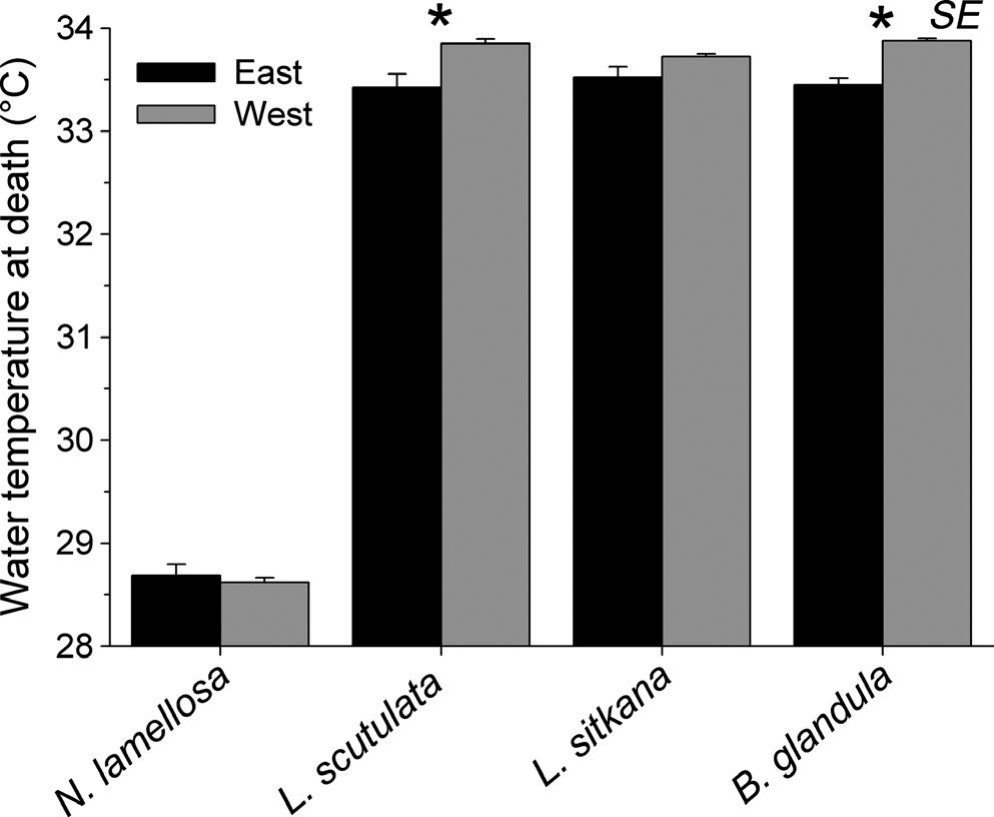
Water temperature at death (TAD) for east and west coast populations of four intertidal invertebrate species on Vancouver Island (*n* = 3 sites per coast). * indicates a significant difference between populations

Intraspecific variation in water temperature tolerance was detected in 2 species. Water temperature tolerance differed significantly between east and west coast populations of *B. glandula* (Nested ANOVA: *F*_1, 4_ = 9.97, *p* = .034) and *L. scutulata* (Nested ANOVA: *F*_1, 4_ = 9.30, *p* = .045), although in this case it was the west coast populations displaying slightly higher (0.4°C) tolerance thresholds to elevated water temperature than east coast populations (Figure 4). Water temperature tolerance did not differ between east and west coast populations of *N. lamellosa* (Nested ANOVA: *F*_1, 4_ = 0.39, *p* = .566) or *L. sitkana* (Nested ANOVA: *F*_1, 4_ = 2.69, *p* = .177).

Interspecific variation in water temperature tolerance was also significantly related to the upper limit of intertidal distribution of these species on both coasts (Table 4). Here again, the trend was mainly due to *N. lamellosa* being substantially less tolerant and distributed lower in the intertidal zone than the 3 other species (Figure 4). Water temperature tolerance did not exceed 34°C for any of the species.

#### 3.1.3 Salinity tolerance

**FIGURE 5:**
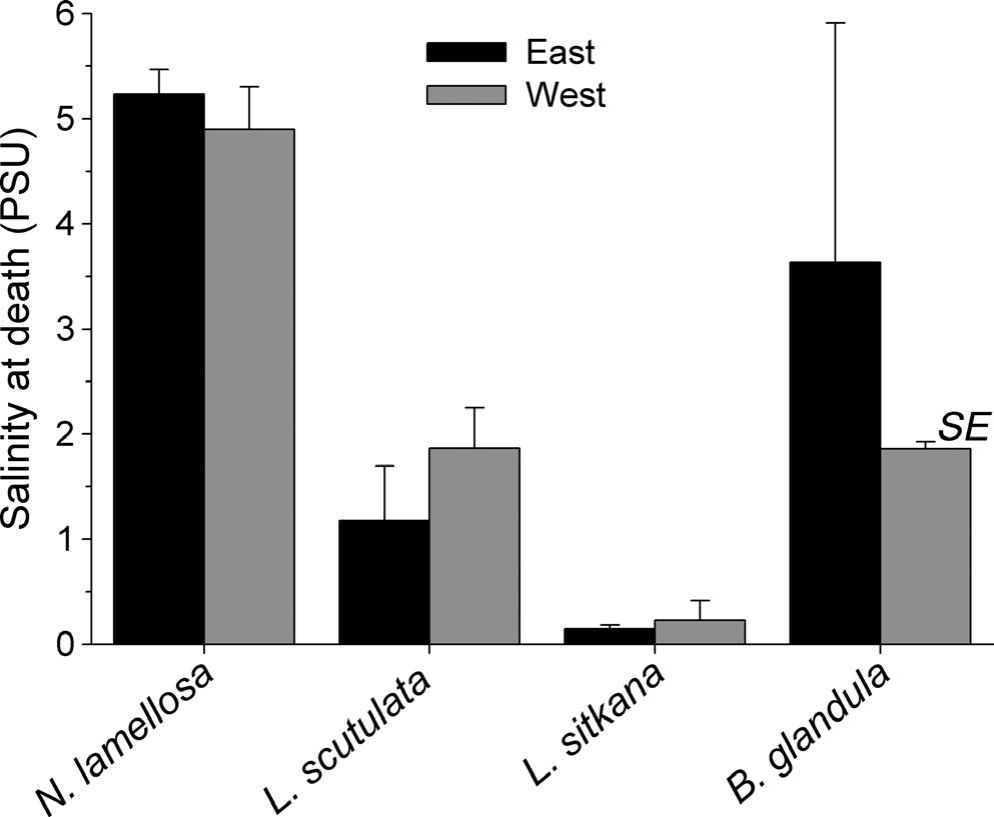
Salinity at death (SAD) for east and west coast populations of four intertidal invertebrate species on Vancouver Island (*n* = 3 sites per coast)

Intraspecific variation in salinity tolerance, between east and west coast populations, was not detected in *N. lamellosa* (Nested ANOVA: *F*_1,4_ = 0.510, *p* = .524), *L. scutulata* (Nested ANOVA: *F*_1,4_ = 1.140, *p* = .351), *L. sitkana* (Nested ANOVA: *F*_1,4_ = 0.175, *p* = .714), or *B. glandula* (Nested ANOVA: *F*_1,4_ = 0.604, *p* = .518). Salinity tolerance differed among species, however, with *N. lamellosa* (*M* = 5.07, *SD* = 0.543) being less tolerant of reduced salinity than *L. sitkana* (*M* = 0.187, *SD* = 0.214)*;* *t*(10) = 20.5, *p* < .001), *L. scutuata* (*M* = 1.52, *SD* = 0.806); (*t*(10) = 8.94, *p* < .001), and *B. glandula* (*M* = 2.62, *SD* = 2.37); (*t*(6) = 2.46, *p* = .049), as seen in Figure 5. Interspecific variation in SAD, however, was not quite significantly related to the upper limit of intertidal distribution (Table 4).

### 3.2 Present‐day tolerance thresholds relative to predicted future conditions

**FIGURE 6:**
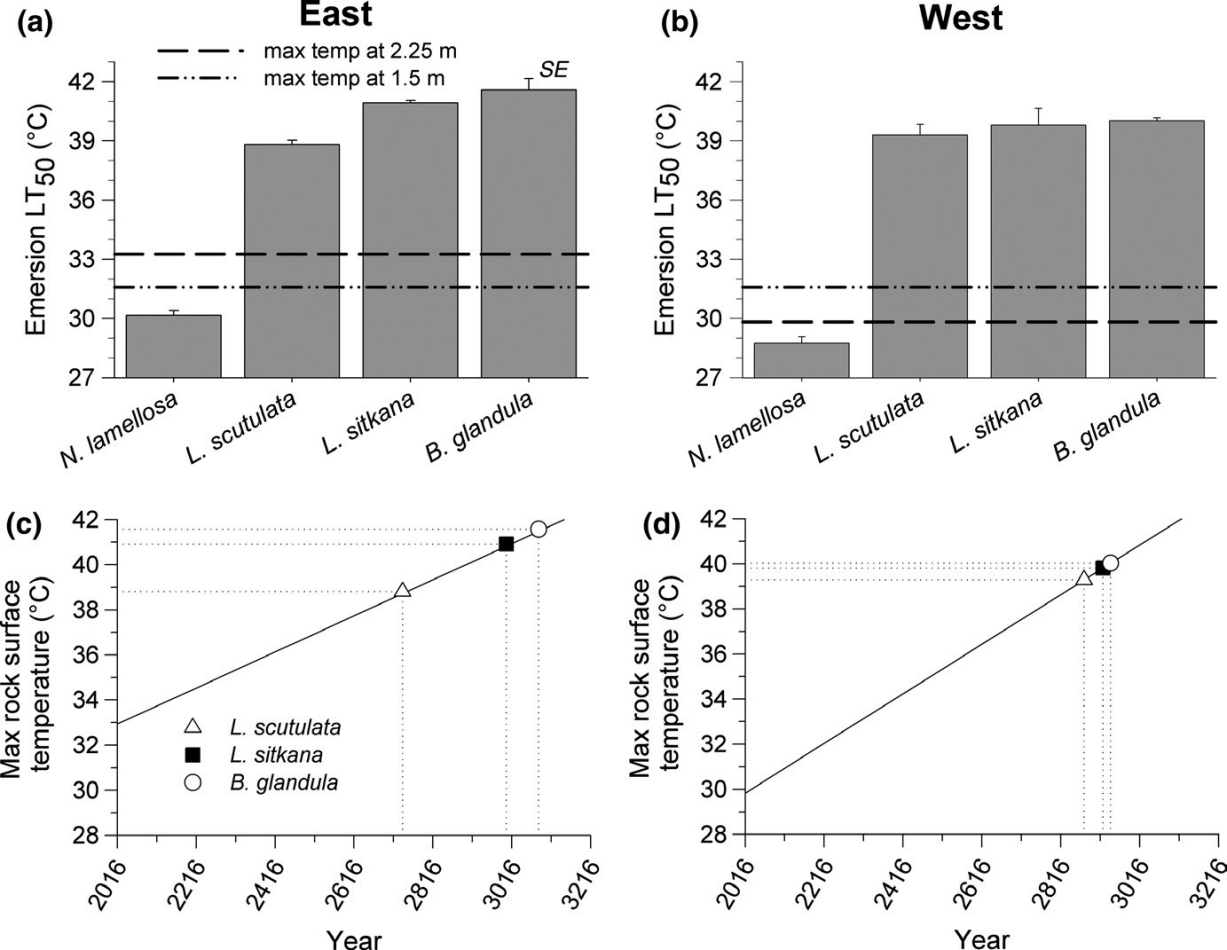
Emersion temperature tolerance (LT_50_) of east (a, c) and west (b, d) coast populations of four marine invertebrate species relative to maximum summertime intertidal rock surface temperatures at low tide. Emersion temperature tolerance thresholds are shown relative to present‐day maximum rock surface temperatures (a, b); the dashed lines represent the single highest maximum summertime (July–August, 2015 and 2016) rock surface temperature at low tide per coast at 1.5 m and 2.25 m. Predicted trends in maximum summertime rock surface temperature at 2.25 m on each coast (assuming recent rates of change will continue into the future) as well as emersion temperature tolerances of populations on each coast (c, d) reveal the estimated year when extreme rock surface temperatures would reach present‐day LT_50_ values, excluding *Nucella lamellosa* (see text). Present‐day extreme rock surface temperatures were obtained from Iwabuchi and Gosselin (2019); extrapolations of those temperatures into the future were based on predicted rates of change in air temperature on each coast by White et al. (2016)

For the 3 upper intertidal species examined herein, emersion LT_50_ values for east and west coast populations were substantially higher than the warmest emersion temperature recorded at 2.25 m on the respective coast (Figure 6a,b). The temperature tolerance thresholds of east coast populations of upper intertidal species were 5.6–8.3°C higher than the highest rock surface temperature recorded on the east coast, while west coast temperature tolerances were 9.5–10.2°C higher than the warmest rock surface temperature recorded on that coast. In contrast, east and west coast populations of the low intertidal species *N. lamellosa* had emersion LT_50_ values that were 1.4 and 2.8°C lower, respectively, than present‐day highest substratum temperatures reported at 1.5 m on either coast (Figure 6a,b). Finally, if substratum temperature increases at the same rate as summertime air temperature (i.e., 0.8°C per century on the east coast and 1.1°C per century on the west coast, White et al., 2016); then, it would take several centuries before predicted maximum emersion temperatures reach present‐day LT_50_ values of east and west coast populations of the 3 upper intertidal species (Figure 6c,d). No such calculations were made for *N. lamellosa*, as emersion temperature tolerances of this species are already exceeded by present‐day maximum temperatures of exposed rock surfaces at 1.5 m.

**FIGURE 7:**
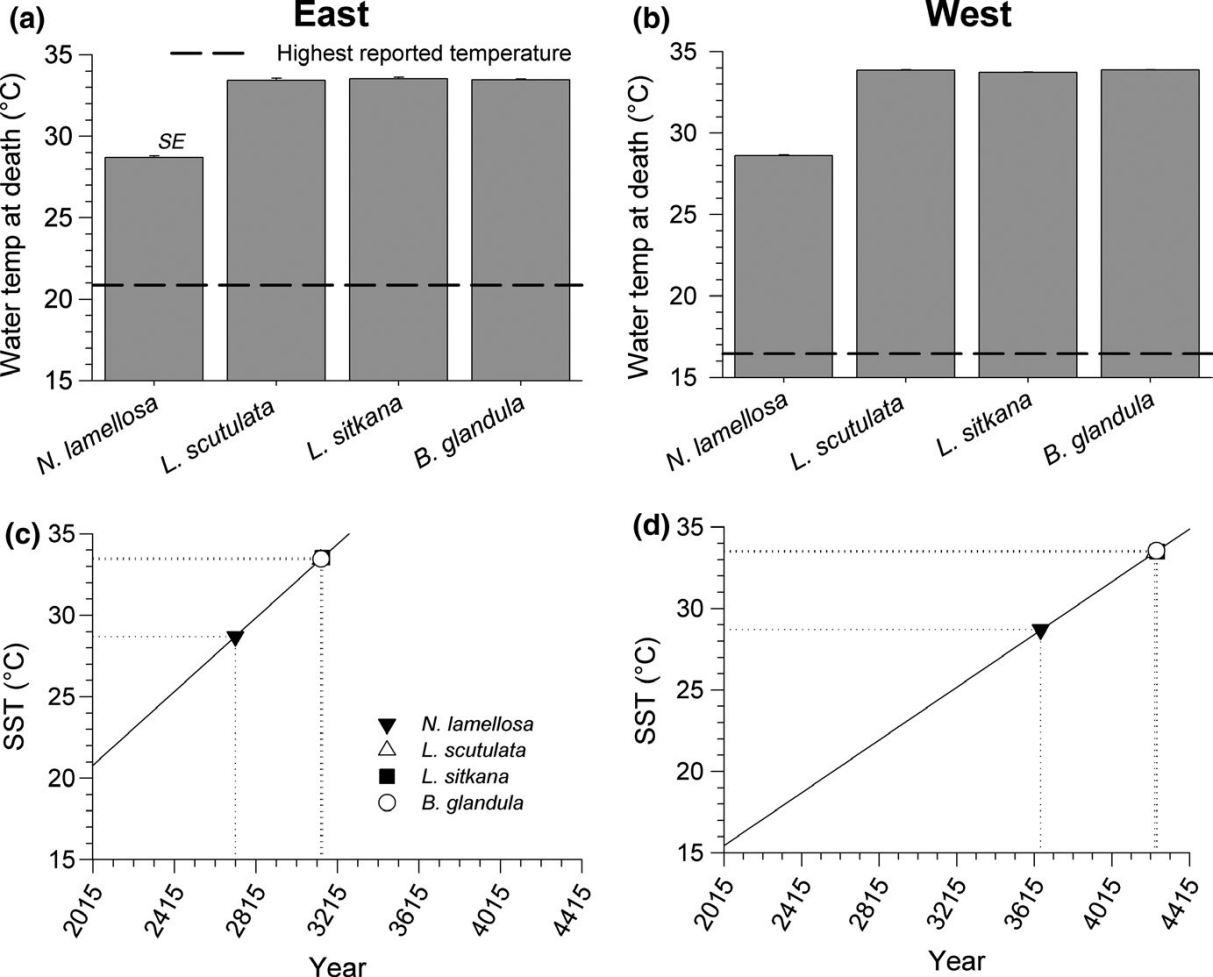
Immersion temperature tolerance (water temperature at death) of east (a, c) and west (b, d) coast populations of four marine invertebrate species relative to maximum summertime sea surface temperature. Immersion temperature tolerance thresholds are shown relative to the maximum summertime sea surface temperatures recorded by near‐shore monitoring stations on each coast (*n* = 2 stations per coast) (a, b); the dashed lines represent the single highest maximum summertime (July–August, 1935–2016) sea surface temperature recorded on each coast. Predicted trends in maximum summertime sea surface temperature on each coast (assuming recent rates of change will continue into the future) as well as immersion temperature tolerances of populations of each coast (c, d) reveal the estimated year when maximum summertime sea surface temperatures would reach present‐day water temperature at death values. Present‐day and future temperature predictions are based on Iwabuchi and Gosselin (2019)

Present‐day water temperature tolerances of east and west coast populations in all 4 species were considerably greater than the warmest extreme SST recorded on each coast in July and August from 2006 to 2016 (Figure 7a,b). East coast populations displayed temperature tolerance thresholds that were 7.8–12.7°C greater than the highest reported extreme SST on the east coast, and tolerance thresholds of west coast populations were 12.1–17.4°C greater than the highest reported SST on that coast. Although maximum summertime SSTs are predicted to become progressively warmer in the future on both coasts of Vancouver Island (Iwabuchi & Gosselin, 2019), maximum SSTs are not expected to match present‐day acute immersion temperature tolerance of any of the 4 species for several hundred years (Figure 7c,d). However, the higher present‐day SSTs on the east coast, coupled with the current rate of increase in SST on the east coast, may create conditions that threaten east coast population persistence sooner than west coast population persistence.

**FIGURE 8:**
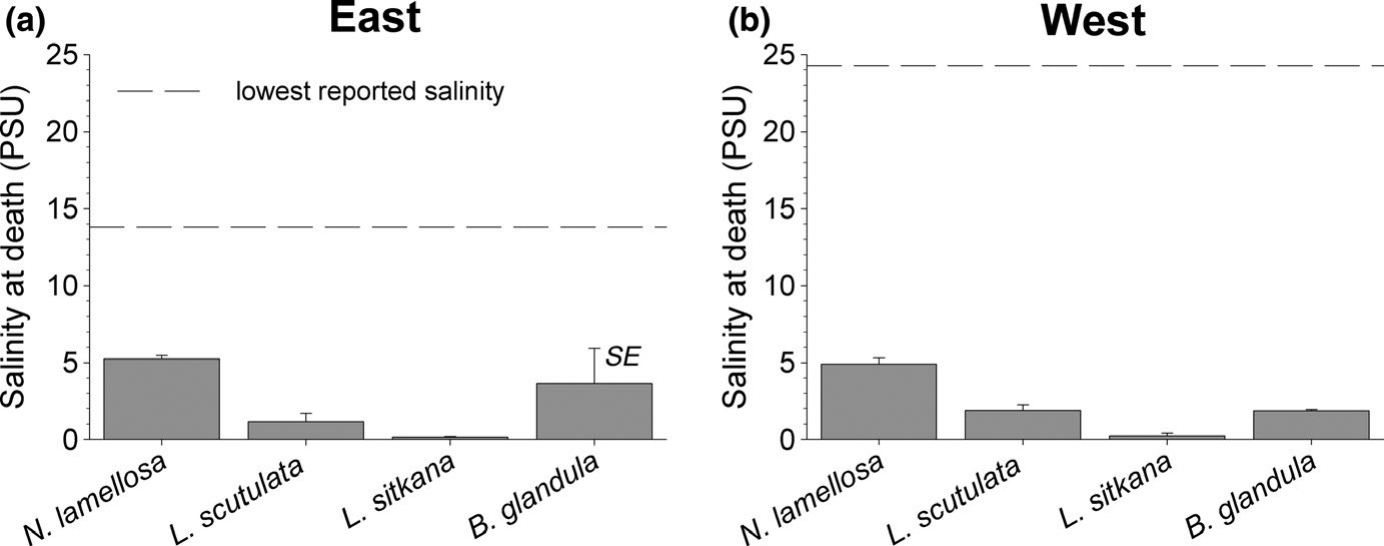
Salinity tolerance (salinity at death) of east (a) and west (b) coast populations of four marine invertebrate species (this study) relative to the lowest salinities recorded by near‐shore monitoring stations on each coast (*n* = 2 stations per coast); the dashed lines represent the single lowest sea surface salinity on the east between June and July, and the west between January and February between 2006 and 2016 (Iwabuchi & Gosselin, 2019)

Populations of all 4 species were able to tolerate acute exposure to salinities substantially lower than the lowest SSS conditions recorded on either coast from 2006 to 2016 (Figure 8a,b). Upper intertidal species were the most tolerant of low salinities, with present‐day salinity tolerance thresholds of east coast populations enabling them to withstand salinity conditions 10.2–13.7 PSU lower than the single lowest SSS presently occurring on the east coast, and west coast populations tolerating acute exposure to salinity conditions 22.4–24.0 PSU lower than the single lowest SSS reported for that coast. Although not quite as tolerant of low salinities as the upper intertidal species, *N. lamellosa* could withstand acute exposure to SSS conditions 8.6 (east) and 19.4 (west) PSU lower than the lowest SSS conditions presently experienced on each respective coast (Figure 8a,b). Given the long‐term trend of increasing minimum SSS on the east coast and the absence of a trend on the west coast since 1935 (i.e., no detectable change in minimum SSS, Iwabuchi & Gosselin, 2019), there is no indication that yearly minimum SSS conditions will become more stressful for the populations studied here for the foreseeable future.

## 4 DISCUSSION

### 4.1 Extent of interpopulation variation in tolerance thresholds

Populations of marine invertebrates living on the east and west coasts of Vancouver Island have been exposed to distinct extremes in SST and SSS conditions at least since monitoring started in 1935, and probably for considerably longer (Iwabuchi & Gosselin, 2019). Intertidal species inhabiting these two coasts also experience different extreme emersion temperatures during the warmest time of the year (Iwabuchi & Gosselin, 2019). Given the early age at first reproduction in these species (1–4 years), east and west coast populations will have experienced these distinct environmental conditions over many generations, providing opportunity for divergent trends in tolerance thresholds. The measures of temperature and salinity tolerance thresholds reported herein, however, suggest a very modest degree of divergence among populations in acute tolerance of extreme temperature and salinity conditions.

The finding that best supported the local adaptation hypothesis was the difference between east and west coast populations in acute tolerance to elevated emersion temperatures in two species, *B. glandula* and *N. lamellosa,* with east coast populations of these species displaying higher emersion temperature tolerance than west coast populations. These higher tolerance thresholds of the east coast populations are consistent with the higher summertime (June–July) rock surface temperatures documented on this coast relative to the west coast. However, summertime rock surface temperatures at low tide were 4.2°C warmer on the east coast (Iwabuchi & Gosselin, 2019), whereas tolerance thresholds to elevated emersion temperatures were ≤1.5°C higher in east coast populations of these species. In addition, no divergence in emersion temperature tolerance was detected between east and west coast populations of the 2 other species, *L. sitkana* and *L. scutulata*. Consequently, emersion temperature tolerance in these 4 species suggests modest or no local adaptation or phenotypic variation between these populations.

East coast populations of intertidal organisms also experience July and August seawater temperatures that are on average 5.2°C warmer than on the west coast (Iwabuchi & Gosselin, 2019). This historical difference in summer SST, however, did not lead to corresponding differences in tolerance of acute exposure to elevated water temperature. Tolerance thresholds to elevated seawater temperature differed between east and west coast populations only in 2 species, *B. glandula* and *L. scutulata,* and these differences were not consistent with summertime SST on those coasts; east coast populations of these two species were less tolerant of elevated SST than west coast populations.

Although SSS fluctuates seasonally on both coasts of Vancouver Island (Pickard & McLeod, 1953), the SSS on the east coast drops substantially lower (8.7 PSU) than on the west coast each year (Iwabuchi & Gosselin, 2019). Accordingly, it was hypothesized that east coast populations of intertidal invertebrates would be more tolerant of reduced salinity than west coast populations of the same species. That, however, was not the case; east and west coast populations of each species had similar tolerance thresholds to low SSS. This lack of interpopulation differences contrasts with previous reports that low‐salinity tolerance can become locally adapted in populations of benthic invertebrates, as shown in *L. sitkana, L. scutulata*, and *Littorina subrotunda* (Sokolova & Boulding, 2004; Yamada, 1989). The lack of divergence in salinity tolerance in the present study may be an indication that SSS is not the most important cause of salinity stress in these populations. Rather, salinity tolerance may be determined mainly by exposure to heavy rainfall during low tide emersion, directly exposing these animals to freshwater for several hours (Dong, Han, & Huang, 2014). The large volume of seasonal rainfall experienced throughout the Pacific Northwest (Thomson, 1981; Tully & Dodimead, 1957) would cause frequent exposure of intertidal animals on both coasts of Vancouver Island to fully freshwater conditions at low tide, causing them to develop similar salinity tolerance thresholds. If so, this would be an important consideration given that future increases in precipitation are predicted for the North Pacific region (IPCC, 2014).

### 4.2 Dispersal ability

This study included 2 species with dispersing planktonic larvae (*B. glandula, L. scutulata*) as well as 2 species with direct development and thus limited dispersal capabilities (*N. lamellosa, L. sitkana*). Although it has been suggested that gene flow might be more restricted in direct‐developing than in planktonic‐dispersing species (Yamada, 1989), leading to greater interpopulation divergence in species with direct development, the findings of the present study, which tested animals from a single generation, did not support that hypothesis. Divergence between east and west coast populations in tolerance to elevated emersion temperature was similar in *N. lamellosa* (1.4°C; direct development) and *B. glandula* (1.5°C; planktonic), and there was no divergence between populations *of L. sitkana* (direct‐developing) or between populations of *L. scutulata* (planktonic). As for immersion temperature tolerance, divergence in tolerance thresholds only occurred in species with planktonic development. Finally, there was no evidence of divergence in tolerance to reduced salinity in either direct‐developing or planktonic‐dispersing species. While these findings are inconsistent with the postulate that direct developers have an increased potential for local adaptation relative to species with planktonic development (Chevin, Lande, & Mace, 2010; Endler, 1977; Hellberg, 1996; Sanford & Kelly, 2011; Yamada, 1989), the present findings add to a growing body of evidence suggesting local adaption is equally common in direct developers and planktonic dispersers (Sotka, 2012).

### 4.3 Present‐day tolerance thresholds vs present and future conditions

Temperature and salinity conditions are not equally stressful year‐round; rather, stress induced by these factors peaks during a limited time of year when conditions reach extreme levels; accordingly, the present analysis focused on the most extreme conditions occurring locally each year rather than use annual or seasonal averages. For populations on each coast, present‐day immersion TAD values were all substantially higher than the warmest SST recently recorded on the respective coast, SAD values were lower than the lowest SSS, and emersion temperature tolerance thresholds of 3 of the 4 species were greater than the warmest emersion temperature on each coast. These findings indicate the populations are not living close to the edge of their upper thermal limits (Sanford & Kelly, 2011), at least in terms of tolerance of acute conditions, and that acute exposure to the most extreme heat and low‐salinity conditions presently occurring on both costs is not a threat to their persistence. The only exception to this was *N. lamellosa,* in which emersion temperature tolerance was lower than present‐day maximum rock surface temperatures, seeming to suggest that *N. lamellosa* populations should not be able to persist at these sites. However, individual *N. lamellosa* position themselves in crevices or under rocks or algae during low tide (pers. obs.), where thermal stress can be substantially lower than on nearby exposed rock surfaces (Garrity, 1984). This would explain why *N. lamellosa* is almost exclusively found in cryptic microhabitats at low tide and suggests the persistence of *N. lamellosa* at a given site is likely dependent on availability of these cryptic microhabitats.

Although the exact rates of change in climate‐related parameters in the future have yet to be determined and will partly depend on future production of greenhouse gasses (IPCC, 2014), long‐term monitoring of SST and SSS along the coasts of Vancouver Island has revealed consistent, linear rates of change in these parameters since monitoring began in 1935 (Iwabuchi & Gosselin, 2019). Those rates are thus likely to closely approximate rates of change in SST and SSS for the near future, and were therefore used as best available estimators of rates of change when predicting future conditions. On Vancouver Island, the yearly minimum SSS has been increasing on the east coast since 1935, thus becoming less stressful, while minimum SSS conditions on the west coast have not changed (Iwabuchi & Gosselin, 2019). Over the same time period, peak summertime SST has been increasing linearly on both coasts and so is expected to become increasingly stressful in the future (Iwabuchi & Gosselin, 2019). Furthermore, maximum substratum temperature in the intertidal zone during low tide emersion is also expected to increase along both coasts into the future (Iwabuchi & Gosselin, 2019). However, even if tolerance thresholds of these intertidal populations were to remain unchanged, future extreme levels of these abiotic factors are not likely to overwhelm present‐day acute tolerance thresholds for quite some time. Thus, despite a seemingly limited capacity for evolution of acute tolerance thresholds, exposure to seasonal acute temperature stress is not expected to be an immediate threat to the persistence of these populations. Nevertheless, our predictions suggest that increasing emersion temperature in the future would become a threat to the persistence of all these populations sooner than acute stress from extreme levels of SST or SSS.

### 4.4 Implications for population persistence

The persistence of coastal populations of marine organisms faced with increasing abiotic stress will depend on overcoming three types of challenges: (a) periodic acute exposure to extreme levels of stressors, (b) chronic exposure to elevated levels of stressors, and (c) indirect effects caused by impacts of the stressors on other parts of the community. The present study examined the first of these challenges and revealed that acute exposure to extreme levels of 3 climate parameters (elevated substratum temperature and SST, and reduced salinity) do not appear to be a threat to the persistence of these species on Vancouver Island in the near future. However, the acute tolerances of larval and early benthic phases should also be taken into consideration when predicting population persistence, as different stages of life can display different tolerance thresholds (Hamilton & Gosselin, 2020). In addition, the persistence of populations in a given region also depends on whether individuals can survive chronic (i.e., long‐term) exposure to sublethal climate‐related stressors, as even moderate levels of climate‐related stress can affect the performance of organisms if they are subjected to these conditions for prolonged periods (Whiteley & Mackenzie, 2016). Exposure to increased temperature conditions for extended periods can negatively affect intertidal animals, such as causing decreased foraging activity and growth rate (Pincebourde, Sanford, & Helmuth, 2008) and reducing upper tolerance limits (Nguyen et al., 2011; Sorte et al., 2011). As emersion temperature increases on the coasts of Vancouver Island, this parameter will likely impose increased levels of chronic stress on populations well into the future. More work on chronic effects, especially with regards to heat stress, is needed, as the implications of chronic effects for the persistence of coastal invertebrate populations are underrepresented in the literature relative to studies focusing on marine vertebrates or terrestrial biota.

Population persistence also depends on impacts of climate‐related stressors on other parts of the community, which then have secondary effects on other species (Harley et al., 2006; Kordas, Harley, & O’Connor, 2011). Although a population may be sufficiently tolerant of abiotic conditions to withstand acute and chronic exposure to the climate conditions of a given area, the population may still be at risk from cascading community level changes that occur when less tolerant organisms are affected by changing climate conditions (Helmuth, Mieszkowska, Moore, & Hawkins, 2013). These indirect effects of climate change have been demonstrated to negatively impact marine ecosystems in a variety of ways, including disruptions to food webs (Ainsworth et al., 2011; Hoegh‐Guldberg & Bruno, 2010; Johnson et al., 2011), increased predation pressure (Harley, 2011), altered interactions with competing species (Hawkins et al., 2008; Kordas et al., 2011), changes to community composition (Sagarin, Barry, Gilman, & Baxter, 1999; Southward, Hawkins, & Burrows, 1995) or increased prevalence of diseases (Harvell et al., 2002; Hoegh‐Guldberg & Bruno, 2010) and parasites (Poulin & Mouritsen, 2006). Although indirect effects could be major determinants of population persistence, there is presently limited information regarding the effects of single indirect stressors, and indirect effects of combinations of climate stressors are even less well understood.

Of the above 3 types of climate‐related challenges, the most immediate threat to population persistence could be acute effects, as a single extreme event could extirpate vulnerable populations; thus, the focus of this study is on acute effects. In that regard, the likelihood of persistence of coastal populations will be enhanced if they are capable of adjusting their tolerance thresholds over time (Bennett et al., 2015; Knight, 2010; Somero, 2010). There are concerns, however, that the rate of change in tolerance thresholds may not be fast enough to keep pace with climate change (Henson et al., 2017). The present study revealed minimal divergence in acute tolerance thresholds between east and west coast populations, supporting the hypothesis that tolerance thresholds to extreme conditions change very slowly in these species. Faced with a warming environment, a slow rate of change in tolerance thresholds could lead to extirpation of a population in the near future if the population has narrow safety margins (i.e., tolerance thresholds only slightly higher than the most stressful conditions presently occurring in the inhabited region; Bennett et al., 2015). However, the populations examined herein all had large acute thermal and salinity safety margins relative to the most extreme conditions in their environment, and present‐day acute tolerance thresholds are predicted to remain higher than future extreme conditions for an extended period of time, likely a few centuries; this finding suggests a slow rate of change in tolerance thresholds might be sufficient for these populations to overcome future increases in acute temperature conditions.

## CONFLICT OF INTEREST

None declared.

## AUTHOR CONTRIBUTIONS

**Brianna L. Iwabuchi:** Data curation (lead); formal analysis (lead); funding acquisition (supporting); methodology (equal); writing‐original draft (lead); writing–review and editing (equal). **Louis A. Gosselin:** Conceptualization (lead); data curation (supporting); formal analysis (supporting); funding acquisition (lead); methodology (equal); supervision (lead); writing–original draft (supporting); writing–review and editing (equal).

## Supporting information

Appendix S1

## Data Availability

Data available from the Dryad data repository (https://doi.org/10.5061/dryad.x95x69pfm).
