# Supplementary material for: Implications of acute temperature and salinity tolerance thresholds for the persistence of intertidal invertebrate populations experiencing climate change
Source: Ecol Evol. 2020 Jun 25;10(14):7739–54. doi: 10.1002/ece3.6498 (PMC7391333; doi:10.1002/ece3.6498)
Supplement: Supplementary file 1 — Appendix S1 [file ECE3-10-7739-s001.docx]

**Appendix A.** Health assessment of animals

The health of the snail *N. lamellosa* was determined by first observing their active state immediately after emersion. Those individuals adhered to the walls of the cage using their muscular foot were deemed healthy, while those that were unattached required further testing using a blunt probe to evoke a response (i.e. movement or retraction of muscular foot and/or siphon) after touching the muscular foot, siphon or operculum as appropriate. If no response was elicited via probe, fine-tipped tweezers were used to gently probe under the operculum. When no responses were prompted, the individual was reported as deceased and removed from the experiment.

Determining the health of *L. sitkana* and *L. scutulata* was similar to the procedures used with *N. lamellosa*; immediately after emersion those individuals who were attached to the walls of the cage using their muscular foot were deemed healthy, while those who were unattached required further testing. Unattached individuals were submersed into a shallow well of sea water for one min and monitored for responses (i.e. emergence from shell and/ or attachment of foot to dish), before using a blunt probe to determine their condition. If no responses occurred after the course of these two procedures the individual was deemed deceased and removed from the experiment.

In the case of *B. glandula,* opercular plates were tapped or gently depressed using a fine-tipped probe to determine if an individual was alive and responsive. Deceased individuals were unable to hold their opercular plates in-situ and would be cleaned from the stone.

**Appendix B.** Preliminary emersion temperature tolerance experiments

Collections of animals were made from east and west coast sites on 2 July 2015 (Table B1). All animals were held for 48 h in trays containing aerated seawater filtered to 200 µm, at 15.0 – 7.5 °C and 30 – 32 PSU. At the onset of the preliminary emersion temperature experiment, cages containing animals were removed from the holding tanks and residual water was blot-dried from the animals and cages. Next, the replicate cages were placed in either air-tight plastic bags or containers that retained high levels of relative humidity and thus minimized desiccation stress throughout the experiment. To ensure high relative humidity, three 4 x 4 cm paper towels saturated with seawater were also added to each bag or container. At the time of sealing the containers, just before moving these to the temperature treatment incubators, sensors (iButton® model DS1923 humidity loggers) inside the containers indicated relative humidity was at least 80%; relative humidity then increased over the following 1 – 2 h in all containers, stabilizing at levels of 90 – 98%. The bags and containers were transferred into temperature-controlled incubators, pre-set to the desired temperature treatment (Table B1).

**Table B1.** Preliminary emersion temperature tolerance experimental design summary per species

| **emersion temperature** | | | | |
| --- | --- | --- | --- | --- |
| Species | Replicate cages per site (n = 6) | Number of individuals per cage | Total number of individuals used in experiment | air temperature treatments (°C) |
| *Nucella lamellosa* | 12 | 5 | 60 | 25, 33, 40 |
| *Balanus glandula* | 12 | 10 | 120 | 25, 33, 40 |
| *Littorina sitkana* | 12 | 10 | 120 | 25, 33, 40 |
| *Littorina scutulata* | 12 | 10 | 120 | 25, 33, 40 |

To determine the range of air temperatures necessary to induce 0 – 100 % mortality within animals, cages with animals were exposed to a particular air temperature for a 12 h duration. After the 12 h treatment, cages or rocks were submerged in aerated seawater between 30 – 32 PSU and held at 17 °C for a 12 h recovery period. Finally, individuals were examined for mortality as described in Appendix A.

**Appendix C.** Preliminary water temperature tolerance experiments

Collections of animals were made from east and west coast sites on 17 July 2015, respectively (Table B1). All animals were held for 48 h in trays containing aerated seawater filtered to 200 µm, at 15.0 – 17.5 °C and 30 – 32 PSU. At the onset of preliminary water temperature tolerance experiments cages were removed from the holding tanks, and distributed amongst aerated experimental tanks, covered with lids and containing 30 – 32 PSU, 200 µ filtered seawater, pre-heated to a desired temperature treatment.

**Table C1.** Preliminary water temperature tolerance experimental design summary per species

| **Water temperature** | | | | |
| --- | --- | --- | --- | --- |
| Species | Replicate cages per site (n = 6) | Number of individuals per cage | Total number of individuals used in experiment | Water temperature treatments (°C) |
| *Nucella lamellosa* | 12 | 5 | 60 | 25, 33 |
| *Balanus glandula* | 12 | 10 | 120 | 25, 33, 40 |
| *Littorina sitkana* | 12 | 7 | 84 | 25, 33, 40 |
| *Littorina scutulata* | 12 | 7 | 84 | 25, 33, 40 |

To determine the range of water temperatures necessary to induce 0 – 100 % mortality within animals, they were immersed at a particular seawater temperature for 24 h, followed by a recovery period immersed in 30 – 32 PSU, 200 µ filtered seawater at 17 °C, and then examined during a 4 h morality check wherein they were exposed to air temperature conditions between 20 – 22 °C (Appendix A). Surviving individuals were then placed in the next warmer temperature treatment (Table B1) for 24 h and was repeated until 100 % mortality was experienced.

**Appendix D.** Upper limits of vertical distribution for east and west coast populations

For the purposes of this study, intertidal species were sampled from their upper limits of their vertical distribution on the east and west coasts of Vancouver Island (Table D1).

| **Table D1**. Upper limit of vertical distribution for intertidal populations on east and west coasts of Vancouver Island. Values are tidal heights relative to lowest low tide (average ± STD). | | |
| --- | --- | --- |
|  | Upper limit of vertical distribution (m) | |
| Species | East | West |
| *Nucella lamellosa* | 2.02 ± 0.05 | 2.24 ± 0.43 |
| *Littorina scutulata* | 4.59 ± 0.09 | 3.08 ± 0.17 |
| *Littorina sitkana* | 4.60 ± 0.09 | 2.98 ± 0.21 |
| *Balanus glandula* | 4.33 ± 0.13 | 3.27 ± 0.48 |

Note that the maximum tidal height on east and west coasts are 5.2 m and 3.9 m respectively.
